# Ultrafast Dynamics of Spin Current and Electron Temperature in Spintronic Terahertz Emitters

**DOI:** 10.1002/advs.75727

**Published:** 2026-05-15

**Authors:** Yifan Wang, Zuanming Jin, Miao Cai, Zhiqiang Lan, Zhenjie Ge, Zheng Feng, Wei Tan, Alexei V. Balakin, Alexander P. Shkurinov, Yan Peng, Yiming Zhu

**Affiliations:** ^1^ THz Technology Innovation Research Institute THz Spectrum and Imaging Technology Cooperative Innovation Center Shanghai Key Lab of Modern Optical System University of Shanghai for Science and Technology Shanghai P. R. China; ^2^ Microsystem & Terahertz Research Center CAEP Chengdu P. R. China; ^3^ Faculty of Physics Lomonosov Moscow State University Moscow Russia; ^4^ Shanghai Institute of Intelligent Science and Technology Tongji University Shanghai China

**Keywords:** electron–phonon coupling, optical‐pump THz‐probe, spintronic terahertz emitters, ultrafast electron dynamics

## Abstract

Femtosecond laser‐pumped spintronic terahertz (THz) emissions have attracted intense academic interest, thanks to their intrinsic capability to generate ultrafast THz pulses that cover a wide spectral range. To gain deep insight into the interplay between the ultrafast thermal dynamics in ferromagnets and the spin‐to‐charge conversion is critical for advancing this field, yet up to now it has not been fully explored, partially due to a lack of efficient tools to measure these two processes simultaneously. Here, we experimentally employ optical‐pump THz‐probe (OPTP) spectroscopy to promote this study in a typical ferromagnet (Ni_80_Fe_20_) nano‐film. It not only elucidates the electron and lattice temperature dynamics by quantifying the optical‐pump‐induced THz transmission changes, but also provides time‐resolved THz spectral maps to analyze the sub‐picosecond changes, which determines a time lag of 63 ± 8 fs between THz emission and laser excitation. This is an intrinsic parameter constraining the upper frequency limit of spintronic THz emission. Our work deepens the fundamental understanding of ultrafast laser excitation mechanisms for spintronic THz emissions and may offer a new perspective to achieve high‐performance THz emitters.

## Graphical abstract

This work quantifies optical‐pump‐induced THz transmission variations to reveal electron and lattice temperature dynamics and presents time‐resolved THz spectral maps for sub‐picosecond analysis. A 63 ± 8 fs time lag between THz emission and laser excitation is identified, serving as an intrinsic parameter limiting the upper frequency of spintronic THz emission.

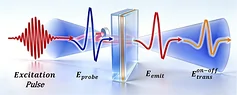

## 1 Introduction

Spintronic terahertz emitters (STEs) typically consist of ferromagnetic (FM) and nonmagnetic (NM) metallic stacks totaling several nm in thickness [1, 2, 3, 4, 5]. STEs are a prime candidate for high‐speed operation on high‐density chips due to their ultrabroadband emission, low cost, scalability, and compatibility with a wide range of material platforms [6, 7, 8, 9, 10]. Additionally, STEs operate with a broad pump wavelength range, making them highly adaptable for table‐top THz spectroscopy [11, 12, 13, 14, 15].

The THz emission mechanism of STEs relies on not only spin‐to‐charge conversion through inverse spin Hall effect (ISHE) or inverse Rashba–Edelstein effect (IREE) [16, 17, 18, 19], but also orbital‐to‐charge conversion through inverse orbital Hall effect (IOHE) or inverse orbital Rashba–Edelstein effect (IOREE) [20, 21, 22]. Upon irradiating the STE with a laser pulse, the electrons and lattice within the metallic layers undergo synchronized thermal excitation. On the one hand, optically excited hot electrons transfer energy to phonons, causing rapid relaxation that raises the effective value of phonon temperature and generates hot phonons [23, 24]. Throughout thermalization, high‐energy phonons are reabsorbed by energetic charge carriers. This reduces cooling efficiency under strong photon‐induced excitation and high hot‐carrier density. Since spin current relies on spin transport within the relaxation time window, the phonon bottleneck effect attenuates the amplitude of the spin current, thereby inducing saturation behavior in the radiated THz wave intensity [25]. On the other hand, short pulse intervals cause heat accumulation, which elevates the steady‐state temperature of the STE. This gradual thermal buildup drives atomic interlayer diffusion, the key mechanism behind STE degradation at high repetition rates [26, 27].

For optimizing thermal management, understanding electron and phonon dynamics in the STEs is critical [28, 29, 30, 31]. This involves three key processes after‐excitation: (1) temperature of electron evolution in the initial few picoseconds; (2) electron–phonon coupling kinetics across the ensuing tens of picoseconds temporal window; and (3) thermal transport phenomena emanating from the laser pumping site—undergoing lateral propagation inside the STEs and vertically penetrating into the substrate on longer timescales. To understand these processes, two key challenges must be properly addressed. First, the electron temperature in STEs was previously investigated via reflection‐mode optical‐pump and optical‐probe (OPOP) spectroscopy [32, 33]. The OPOP measurement revealed electron temperature of a W/NiFe/Pt exceeding 1000 K under a pumping fluence condition of ∼1.5 mJ/cm^2^, with relaxation to steady states over hundreds of picoseconds [32]. However, the results were affected by ambiguous thermal electron accumulation induced by the probe beam. Second, the timescales for material heating and spin current generation fall in the range of ∼100 fs, a duration far shorter than the temporal width of a THz‐frequency probing pulse [34, 35]. The potential temporal lag between ultrafast heating and the spin‐charge current conversion awaits further experimental verification [36].

In this work, we overcome these two challenges by using optical pump and THz probe (OPTP) spectroscopy encompassing both THz emission and THz‐probe propagation [37]. First, unlike visible light, THz photons possess low photon energy (on the order of meV), thus avoiding direct single‐photon or multi‐photon excitation within the sample [38, 39, 40, 41, 42]. Second, while strong‐field THz pulses have been demonstrated to induce electron heating, demagnetization, and spin current generation in ferromagnetic layers [43, 44, 45], the THz field strength used in this work is approximately 800 V/cm, which barely perturbs the electronic and spin degree of freedom and prevents ionization damage to the sample. Third, the 500‐Hz low‐repetition‐rate OPTP scheme suppresses cumulative heating from successive pulses, enabling the characterization of electron temperature dynamics induced by a single optical pump pulse. Furthermore, we have demonstrated time‐resolved THz spectral maps to analyze the sub‐picosecond changes in the OPTP experiments, which are related to the microscopic process leading to THz emission. Our OPTP measurements reveal ultrafast laser‐induced excitation dynamics in ferromagnets and highlight their great potential for high‐efficiency and broadband THz emitters.

## 2 Results

**FIGURE 1:**
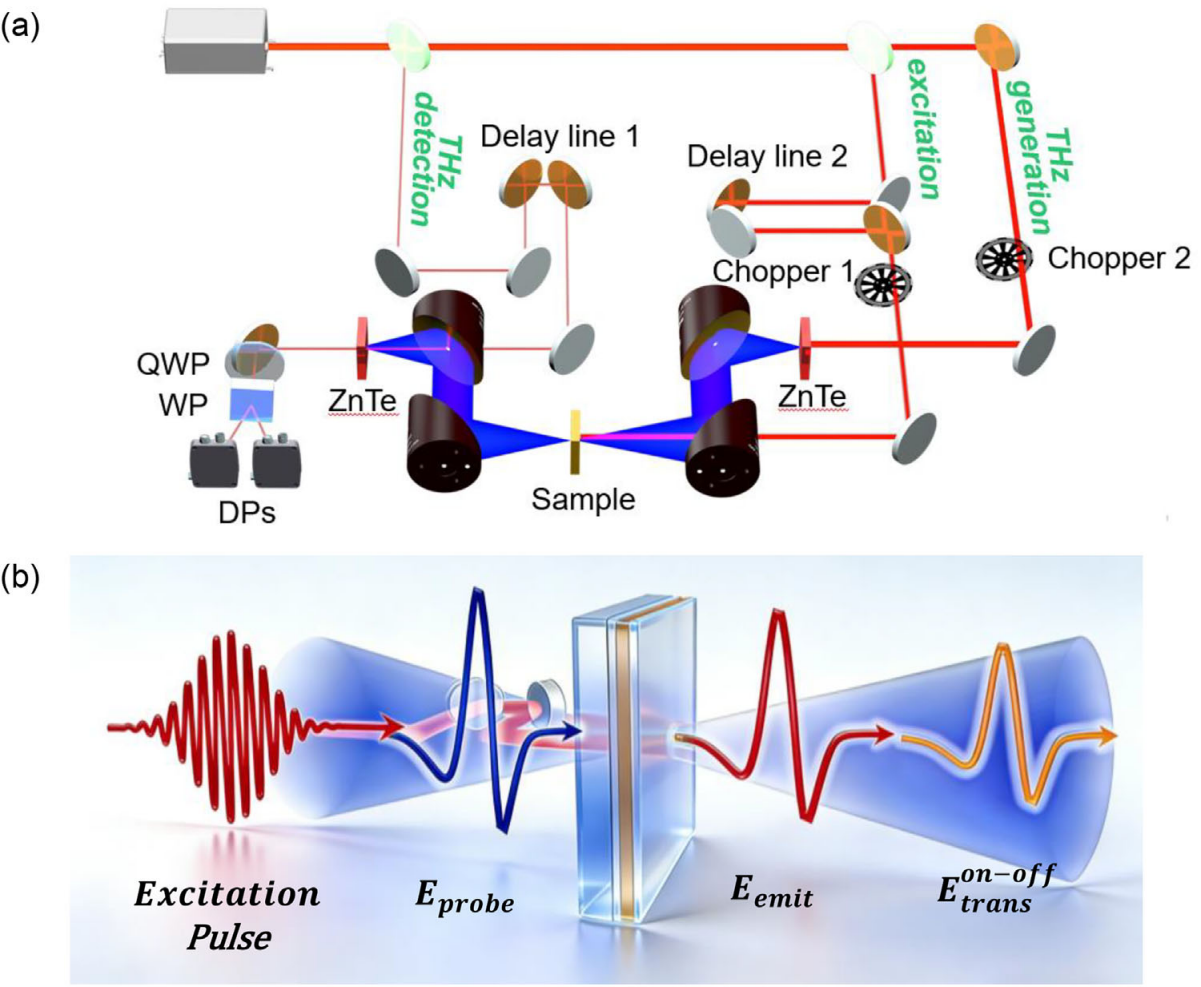
Schematic and spectroscopic characteristics of the OPTP system. (a) Schematic of the OPTP setup. (b) Concept figure: (a) THz probe pulse is injected into the experimental setup. The THz waveform variation $E_{\mathrm{trans}}^{\mathrm{on}-\mathrm{off}}$ under excited vs. non‐excited states is measured, with a detectable THz emission pulse *E*_emit_ from the STE sample.

Our sample consisted of a Ni_80_Fe_20_ (NiFe) film with a thickness of 7 nm deposited on an Al_2_O_3_ substrate, with a 3‐nm‐thick SiO_2_ layer serving as the capping layer (see Section 4). The OPTP spectroscopy is schematically illustrated in Figure 1a, where the optical beam is represented by the red line and the blue region denotes the THz field. OPTP spectroscopy has 1D and 2D operation modes (see Section 4). As shown in Figure 1b, the photoinduced OPTP signal is given by,

(1) $$\Delta E^{\pm H}\;\left(t\right)=E_{\mathrm{emit}}^{\pm H}+\Delta E\left(t\right)$$

where the first term $E_{\mathrm{emit}}^{\pm H}$ corresponds to the THz emission waves generated by the excited NiFe sample, with ± H denoting the direction of the applied magnetic field. The second term denotes the pump‐induced THz transmission change of the THz probe through the sample, $\Delta E\;\left(t\right)=E_{\mathrm{trans}}^{\mathrm{on}}\;-E_{\mathrm{trans}}^{\mathrm{off}}$. $E_{\mathrm{trans}}^{\mathrm{on}}$ and $E_{\mathrm{trans}}^{\mathrm{off}}$ denotes the THz transmission through the sample with and without pumping, respectively. Notably, the magnetic field can modulate THz emission $E_{\mathrm{emit}}^{\pm H}$, whereas the THz transmission change Δ*E*(*t*) remains unaffected by the magnetic field (see Section S1). Thus, the $E_{\mathrm{emit}}^{\pm H}$ and Δ*E* can be distinguished by switching the magnetic fields between +H and − H, expressed as,

(2) $$\Delta E\left(t\right)=\frac{1}{2}\;\left(\Delta E^{+H}\left(t\right)+\Delta E^{-H}\left(t\right)\right)$$

(3) $$E_{\mathrm{emit}}^{\pm H}\left(t\right)=\frac{1}{2}\;\left(\Delta E^{\pm H}\left(t\right)-\Delta E^{\mp H}\left(t\right)\right)$$

**FIGURE 2:**
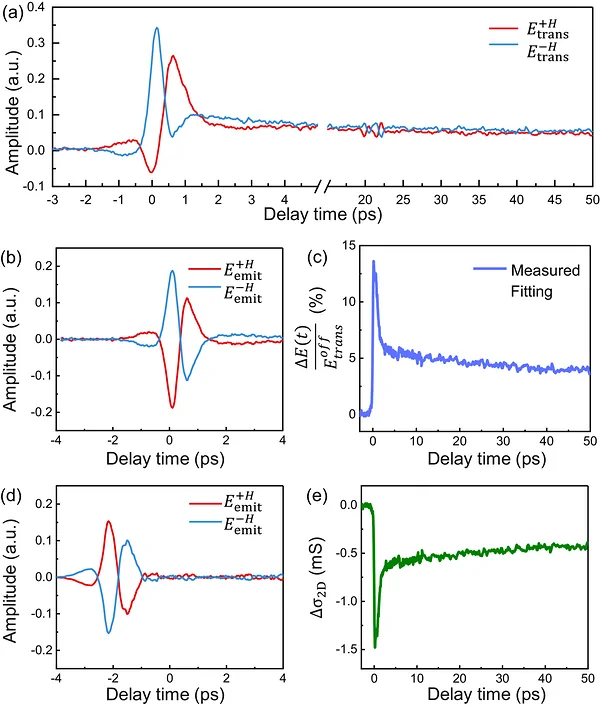
Measured OPTP signal, the reconstructed THz emission, and conductivity dynamics of NiFe sample. (a) THz OPTP curves Δ*E* ^±H^(*t*) of NiFe film was measured under ± H when pumping from the NiFe side. (b) THz emission signals from the sample with ±H fields, obtained via Equation (3). (c) Time‐resolved pump‐induced THz transmission change through the sample, extracted by Equation (2). (d) THz emission from NiFe with flipping the sample. (e) The calculated transient sheet conductivity dynamics Δ*σ*_2D,_ from $\Delta E\left(t\right)/E_{\mathrm{trans}}^{\mathrm{off}}$ in (c).

First, in the 1D operation mode of OPTP, we fix the timing of the electro‐optic sampling probe beam at the THz probe pulse peak position, and scan the Delay Line 2 to change the temporal shift between excitation and THz probe pulses. As shown in Figure 2a, we recorded both the THz electric field transmitted through the 7 nm NiFe thin film and THz emission waveforms from the sample. According to Equations (2) and (3), we obtained the THz emission signals Δ*E* ^±H^(*t*) from the NiFe film under ±H, as illustrated in Figure 2b. In addition, we extracted the time‐resolved THz transmission dynamics Δ*E*, as shown in Figure 2c.

Figure 2b shows the THz electric field generated by the 7 nm NiFe film. The emitted THz waves show polarity reversal with the applied magnetic field, which confirms that they originate from magnetic effects. As shown in Figure 2d, when pump injection is switched from the NiFe film to the Al_2_O_3_ substrate, the THz radiation polarity is reversed. To elucidate the mechanism underlying THz generation in single‐layer NiFe films, we present the following analysis. First, the THz field originating from ultrafast demagnetization (UDM) is given by $E_{\mathrm{dem}}\left(t\right)\propto \frac{\partial^{2}M\left(t\right)}{\partial t^{2}}\cdot \hat{M}\times \hat{z}$, where *M*(*t*) is the time‐dependent magnetization and $\hat{z}$ is the unit vector normal to the film plane [31]. Its polarity is independent of both the film side and the pump propagation direction [46, 47]. Second, previous studies have demonstrated the anomalous Hall effect (AHE) as an important THz generation mechanism [48, 49]. Within the AHE framework, a laser‐induced net backflow longitudinal current *J*_c′_(*t*) can be converted into a transverse current *J*_c_(*t*), which subsequently emits THz radiation: $E_{\mathrm{AHE}}\left(t\right)\propto \frac{\partial }{\partial t}\left[J_{c}\left(t\right)\right]=\frac{\partial }{\partial t}[\theta_{\mathrm{AHE}}\;\cdot J_{c^{\prime }}\left(t\right)\times \hat{M}]$, θ_AHE_ is the anomalous Hall angle [31]. For the Al_2_O_3_/NiFe(7 nm)/SiO_2_ sample, the contribution of AHE to THz emission is found to be 2.56 times that of UDM (see Section S2 for details). Third, a portion of the spin current *J_s_* can be converted into an orbital current *J_L_* = η_*L* − *S*_ *J_s_* [50]. Such spin and orbital currents have been demonstrated to generate transient charge currents *J*_c_ via IREE or IOREE at FM/Ag/Bi or FM/metal‐oxide interfaces, respectively [51, 52]. Although our heterostructure does not contain such interfaces, the possible contributions from IREE and IOREE cannot be completely excluded and deserve further systematic investigation. It should be mentioned that, if a conventional STE were employed in this study, the large amplitude disparity between signals *E*_emit_(*t*) and Δ*E*(*t*) would introduce significant analysis errors. Therefore, a ferromagnetic NiFe film is used as a suitable platform for the study.

Figure 2c shows the positive $\Delta E\left(t\right)/E_{\mathrm{trans}}^{\mathrm{off}}$, corresponding to the real part of the sheet photoconductivity Δσ_2D_(*t*). When the sample absorbs an fs laser pulse, the pulse transfers its energy to the free‐electron plasma, raising the electron gas temperature. Consequently, that increases the electron scattering velocity and reduces conductivity, which yields negative photoconductivity, as shown in Figure 2e. The time‐resolved THz sheet photoconductivity Δσ_2D_(*t*) can be extracted by [53, 54, 55],

(4) $$\Delta \sigma_{2D}=\frac{1+n}{Z_{0}}\;\left(\frac{1}{1+\Delta E_{\mathrm{trans}}/E_{0}}-1\right)$$

where *n* = 3.1 is the refractive index of the Al_2_O_3_ substrate in the THz spectral range, and *Z*_0_ = 377 Ω is the impedance of free space. Substituting the experimentally measured relative transmission variation Δ*E*_trans_/*E*_0_ into this expression yields the real‐valued sheet photoconductivity Δσ_2D_. An increase in THz conductivity leads to a reduction in THz transmittance, signifying that electron‐lattice thermalization drives a decrease in electron temperature. Once thermal equilibrium is established between electrons and the lattice, the entire excited region cools down synchronously.

**FIGURE 3:**
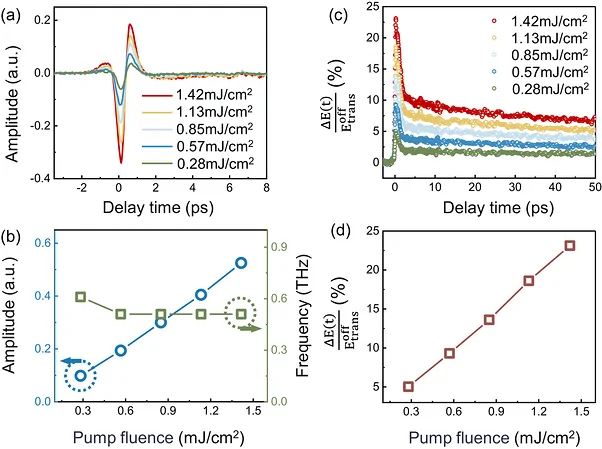
THz emission and carrier relaxation characterizations. (a) THz radiation emitted by a 7 nm thick NiFe film under varied pump fluences. (b) Peak‐to‐peak amplitude and peak frequency of THz emission dependence on the applied pump fluence. (c) The pump fluence is dependent $\frac{\Delta E(t)}{E_{\mathrm{trans}}^{\mathrm{off}}}$ plotted vs. the delay time of optical pumping relative to THz probing pulses. (d) Peak values corresponding to $\frac{\Delta E(t)}{E_{\mathrm{trans}}^{\mathrm{off}}}$ as functions of pump fluence.

To further investigate both THz emission and carrier relaxation dynamics, the OPTP curves of the NiFe film are measured under different pump fluences (see Section S3). Figure 3a shows the waveforms of THz emission $E_{\mathrm{emit}}^{+H}$ with laser excitation varying over the interval of 0.28–1.42 mJ/cm^2^. The corresponding THz spectra are shown in Section S4. As shown in Figure 3b, the peak‐to‐peak amplitudes of the THz signal increase linearly with rising pump fluence. In addition, the peak frequency decreases from 0.61 to 0.51 THz when the laser fluence changes from 0.28 to 1.42 mJ/cm^2^. Since THz emission is highly related to time‐varying magnetization, spectral redshift correlates with slower ultrafast demagnetization in NiFe film excited by higher pump fluence [56].

Time‐resolved carrier dynamic processes under different pump fluences are shown in Figure 3c. The peak value of $\frac{\Delta E(t)}{E_{\mathrm{trans}}^{\mathrm{off}}}$ exhibits a linear dependence on pump fluence, varying from 5%–23% over the pump fluence range of 0.28–1.42 mJ/cm^2^, as shown in Figure 3d. This observation aligns with the enhanced scattering of mobile charge carriers after photoinduced excitation. To clarify the dynamics of $\frac{\Delta E(t)}{E_{\mathrm{trans}}^{\mathrm{off}}}$ or Δσ_2D_(t) with increasing pump fluence, the electron–phonon coupling must be analyzed [57].

After photo‐excitation, the two‐temperature model (TTM), which characterizes the electronic system temperature T_e_ and lattice temperature T_l_, can quantitatively characterize carrier dynamics in metals [58, 59, 60]. When the thickness of metallic FM layers is less than the inelastic mean free path of electrons, the lateral hot‐electron transport is suppressed. Consequently, the energy relaxation depends on electron–phonon coupling instead of hot‐electron diffusion [61, 62]. The electron system's cooling is modeled via coupled differential equations [63],

(5) $$C_{e}\;\frac{dT_{e}}{dt}=-G_{\mathrm{el}}\left(T_{e}-T_{l}\right)+P\left(t\right)-C_{e}\frac{\left(T_{e}-T_{\mathrm{amb}}\right)}{\tau_{\mathrm{th}}}$$

(6) $$C_{l}\;\frac{dT_{l}}{dt}=G_{\mathrm{el}}\;\left(T_{e}-T_{l}\right)$$

where, *C*_e_ and *C*_l_ denote the specific heats of electrons and lattice, respectively. *G*_el_ denotes the electron–phonon coupling constant, a parameter that dictates the magnitude of energy transferred from excited‐state electrons to lattice vibrational modes. *T*_amb_ refers to the ambient environmental temperature. τ_th_ denotes the characteristic heat diffusion time scale. The initial term in Equations (5) and (6) characterizes the electron‐lattice coupling. In Equation (5), the middle term *P*(*t*) represents the excitation source, and the final term characterizes the thermal diffusion process.

**FIGURE 4:**
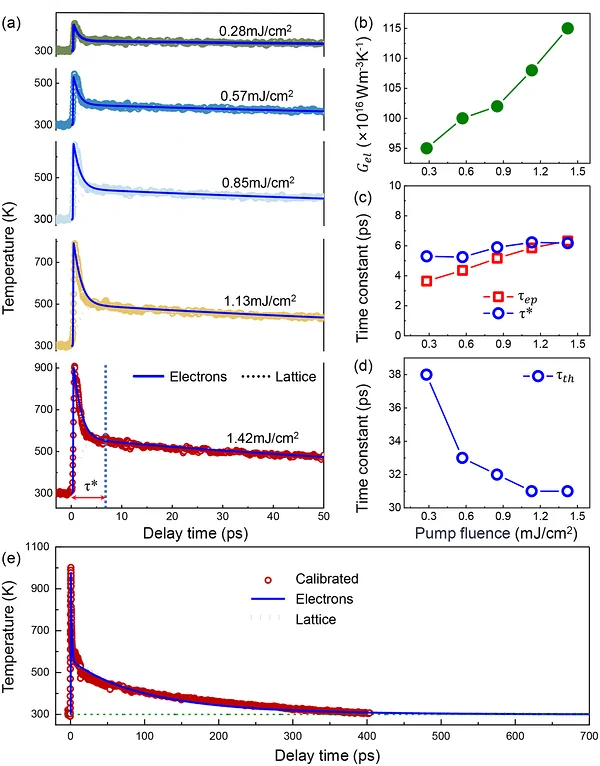
Electron and lattice peak temperature decays: two‐temperature model fitting. (a) The temperature dynamics derived from the THz probe pulse, as shown in Figure 3d, with electron (line) and lattice (dot) temperatures from two‐temperature model fitting. (b) Dependence of the electron–phonon coupling constant *G*_el_ on laser pump fluence (c) Comparison between τ_ep_ (red squares) derived from *G*_el_ extracted via TTM fitting and τ^∗^ (blue circles) obtained from the TTM fitting curves. (d) The heat diffusion time τ_th_ vs. laser pump fluence. (e) An extended temporal window of the temperature dynamics measured at 1.42 mJ/cm^2^.

We further assumed that the electron‐lattice coupling vanished as the electron temperature equilibrated with the lattice temperature. The electron specific heat capacity is temperature dependent, *C*_e_ ═ γ_e_ *T*_e_ with γ_e_═ 3 × 10^3^ J/m^3^/K^2^ [63]. Using the values of Ni [63] and Fe [64], the specific heat capacity of the NiFe sample is estimated as *C*_specific_ ═ 3.8 × 10^6^ J/m^3^/K^1^. Thus, for low pump fluence, the lattice specific heat capacity is *C*_l_ ═ *C*_specific_ (300 *K*) − *C*_e_ (300 *K*) ═  2.9×10^6^ J/m^3^/K^1^. *C*_l_ is lattice‐temperature dependent. For a sample thickness d ═ 7 nm, the surface specific heat capacity of the NiFe film is calculated as *C*_surface_ ═ 2.66 × 10^−6^ J/cm^2^/K^1^. The laser pulse absorptance in our sample is approximately 45%, which is assumed to be uniform across the illuminated area and depth. The measured transmittance, reflectance, and absorptance ratios of the 7 nm NiFe thin film are presented in Section S5. For example, when the absorbed excitation fluence is F ═ 1.42 mJ/cm^2^, the steady‐state temperature is estimated as $T_{\mathrm{NiFe}}^{\mathrm{steay}-\mathrm{state}}=T_{\mathrm{amb}}+F/\;C_{\mathrm{surface}}=546\;K$, which calibrates the temperature scale in Figure 4a.

Figure 4a shows the fits of temperature‐calibrated data for a 7 nm NiFe film using Equations (5) and (6), with blue solid and black dots representing electron and lattice temperature, respectively. It can be seen that electron and lattice temperatures equilibrate within several picoseconds [65, 66]. We use the coupling coefficient between electrons and phonons, *G*_el_, which is derived from Equations (5) and (6), as a scaling factor to fit the experimental results. Notably, the TTM model reproduces our measurements well. The dependences of *G*_el_ and τ_th_ on pump fluence are presented in Figure 4b,d, respectively. Generally, *G*_el_ can be expressed as (see Section 4) [67, 68]:

(7) $$G_{\mathrm{el}}=0.1175{\left(\frac{k_{B}\Theta_{D}e}{\hbar }\right)}^{2}\frac{\rho }{T_{l}}n_{e}^{\frac{4}{3}}$$

where, Θ_D_ is the Debye temperature, a parameter denoting the temperature needed to activate the phonon mode with the maximum frequency, ρ is the resistivity, and *n*_e_ is free electron density. As shown in Figure 4b, the extracted *G*_el_ increases from 95 × 10^16^ to 115 × 10^16^ W/m^3^/K^1^ with increasing pump fluence. The relaxation time of the electron–phonon τ_ep_ can be expressed in terms of *T*_e_ and *G*_el_ [67],

(8) $$\tau_{\mathrm{ep}}\approx \frac{2\gamma_{e}T_{e}}{G_{\mathrm{el}}}$$

where γ_e_═ 3 × 10^3^ J/m^3^/K^2^ and *T*_e_ takes its maximum value. Using these parameters, we calculated τ_ep_ (red squares) for pump fluences ranging from 0.28 to 1.42 mJ/cm^2^, as plotted in Figure 4c. Meanwhile, as indicated by the blue dashed line in Figure 4a, we define *τ* ^∗^ as the time constant at which the electron temperature and phonon temperature derived from TTM fitting converge to the same value. As shown in Figure 4c, the calculated *τ*_e__p_ is in good agreement with *τ* ^∗^ (blue circles) and with the values reported in the literature [69, 70]. Accordingly, we conclude that τ_ep_ represents the characteristic time constant associated with electron–phonon coupling.

Figure 4d shows the extracted heat diffusion time τ_th_ decreases with increasing pump fluence. These values are comparable in magnitude to those reported for Ni (τ_th_≈50 ps) [63], and W/Fe_60_Co_20_B_20_/Pt (τ_th_≈35 ps) [29]. In an effort to characterize the full spatiotemporal evolution of electron and phonon temperatures, we performed a long‐range temporal scan with model fit, as shown in Figure 4e. Our findings reveal that the electron system returns to its baseline state at around 600 ps after photoexcitation, a behavior stemming from lattice thermal diffusion as well as the interfacial thermal resistance of the metal film‐substrate interface. The relaxation of 600 ps identifies a fluence threshold of heat accumulation‐induced damage in the ∼1.67 GHz NiFe film. This value is analogous to 2 GHz threshold observed in W/Fe_60_Co_20_ B_20_/Pt [29].

**FIGURE 5:**
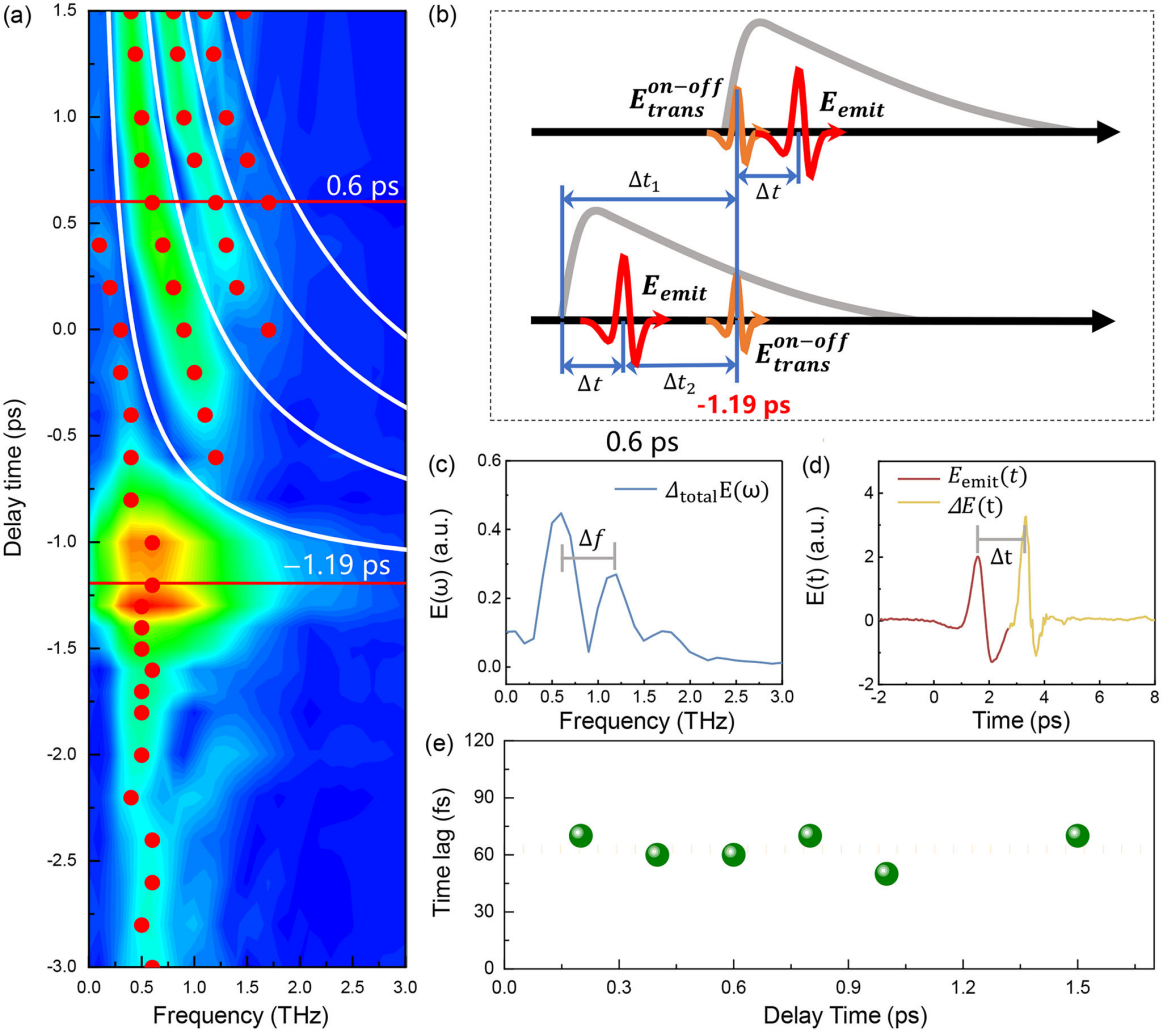
OPTP analysis of NiFe film. (a) 2D spectral map of *E*_emit_ + Δ*E* vs. THz frequency and time delay, when the THz probe is sent into the sample after the excitation. (b) Concept schematic: the measured THz waveform variation $E_{\mathrm{trans}}^{\mathrm{on}-\mathrm{off}}$ (orange) with a detectable THz emission pulse *E*_emit_ (red). The gray curve represents the laser excitation curve. (c) THz spectra and (d) associated THz‐TDS data at +0.6 ps relative time. (e) The time lag between THz emission and laser excitation Δ*t* obtained at different relative delay times.

Finally, we focus on the 2D scan: for each selected Δ*t*_1_, Delay Line 2 is fixed while Delay Line 1 is scanned to map the THz trace *E*(*t*_THz_, Δt_1_), which is then Fourier transformed to obtain $\tilde{E}$(ω, Δ*t*_1_). This method yields a series of $\tilde{E}$(ω, Δ*t*_1_) values across varying optical pump‐THz probe time delays [71]. For our sample, Figure 5a presents the full spectral mapping of *E*_emit_ + Δ*E*(*t*) against delay time and frequency, where red indicators denote the spectral peak positions at distinct time delays Δ*t*_1_. The THz spectra and their corresponding time‐domain spectra for each specific pump delay are presented in Figures S7 and S8 of Section S6. We observe that the presence of THz emission pulses modulates the signal strength of THz probe pulses at the detector site. Accordingly, two key time delays are identified, as shown in Figure 5b. Δ*t*_1_ is the temporal delay of the THz probe relative to the optical excitation pulse. Δ*t*_2_ refers to the time interval of THz emission pulses relative to THz probe pulses. The gray curve represents Δ*E*(*t*), which reflects the time‐dependent scattering rate γ and/or carrier density n. Assuming THz emission occurs Δ*t* after laser excitation, the time interval between them can be obtained as Δ*t*  ═  Δ*t*_2_ − Δ*t*_1_.

As has been theoretically reported previously, the spectral bandwidth reaches its maximum when the temporal centers of the excitation pulse and the probe pulse coincide [40]. Furthermore, a shorter excitation pulse rise time yields a broader spectral bandwidth. Thus, the time point of the widest frequency spectrum can be marked as the moment when the rising process of the laser pulse interacts with the THz detection pulse. As shown in Figure 5a, the relative time position of −1.19 ps is recorded as the position of the THz detection pulse. This relative time position of the THz probe pulse is fixed. In the experiment, the pump beam stepper motor is then moved to the relative time positions of +0.6 ps, giving the temporal delays of the THz probe relative to the optical pump pulse of Δ*t*_1_ = 1.79 ps. The interference patterns of the THz generation and the THz probe pulses at this moment is recorded in Figure 5c. A notable observation is that distinct interference peaks appear across the full spectrum when the probe pulse is introduced after the pump pulse. The interference spectrum shape is governed by the relative amplitudes of the *E*_emit_ and the THz probe pulse Δ*E*(*t*). The spectral dips are confirmed to originate from the destructive interference between *E*_emit_ and Δ*E*(*t*). The frequencies of these dips are determined by the delay time Δ*t*_2_, which matches the predictive formula Δ*t*_2_  ×  f  =  k  +  0.5, (k = ±0, ±1, ±2, …), as illustrated by the white solid curves in Figure 5a. Critical details regarding the temporal gap Δt_2_ of THz radiation and the probe pulse can be derived from the inter‐peak frequency difference Δ*f* of the overall spectrum, with the relationship approximated by Δ*t*_2_ $\approx \frac{1}{\Delta f}$. As shown in Figure 5d, the Δ*t*_2_ is 1.73 ps. On this basis, we calculated the time lag between THz emission and laser excitation, with a value of Δ*t* =  60 fs. Subsequently, we employed the same procedure to calculate the time lag Δt between THz emission and laser excitation at various selected relative delay times. The average time lag was determined to be approximately 63 ± 8 fs, as presented in Figure 5e (see Section S7 for details). To further validate our experimental findings, we performed supplemental measurements on a 7 nm‐thick NiFe sample at an increased pump fluence of 1.98 mJ/cm^2^ (Figure S10) and on a 3 nm‐thick NiFe film at the same pump fluence of 1.42 mJ/cm^2^ (Figure S11). The extracted time lags were 63 ± 5 and 61.7 ± 7.5 fs, respectively. THz emission is observed to occur a finite time after photoexcitation, typically on the order of tens of femtoseconds. The ∼60 fs time lag extracted from 2D measurements on a single layer of NiFe is consistent with the spin‐flip time, an intrinsic material parameter [72]. This result can be contrasted with the characteristic time lag of 120 fs reported by Wang et al. in topological insulator/ferromagnet heterostructures [73].

## 3 Conclusions

In summary, we demonstrate a comprehensive experimental approach to characterize electron and lattice temperature dynamics in NiFe film using optical‐pump THz‐probe measurements. A key advantage over optical probes is that THz pulses do not perturb electron and lattice temperature dynamics, making this technique particularly well‐suited for investigating cumulative heating effects. This method enables the investigation of the pump fluence‐governed optical damage process, revealing a ∼600 ps relaxation time and a 1.67 GHz damage threshold. Furthermore, findings from our OPTP measurements serve as critical reference data for the time‐domain dynamics of ultrafast laser excitation in NiFe. We determine a time lag between THz emission and laser excitation, a parameter crucial for optimizing the bandwidth of STE. Looking forward, future studies into substrate effects and pump spot size could help quantify heat accumulation and clarify the cooling pathways. This experimental work not only deepens the fundamental understanding of ultrafast laser excitation mechanisms but also provides an experimental basis for optimizing the thermal management of STE and fabricating high‐performance THz emitters suitable for advanced THz imaging and focusing systems [74].

## 4 Methods

### 4.1 Sample Preparation

Ni_80_Fe_20_ (NiFe) thin films were grown on the Al_2_O_3_ substrates using an ultrahigh vacuum magnetron sputtering system at room temperature. The sputtering chamber was maintained at a base pressure of 5 × 10^−^^6^ Pa, while the working argon (Ar) gas pressure was set to 0.4 Pa during the deposition process. To mitigate oxidation of the NiFe films, a 3‐nm‐thick SiO_2_ capping layer was deposited as a protective coating.

### 4.2 Optical Pump THz Probe Spectroscopy

The OPTP spectroscopy was performed using a Ti:sapphire regenerative amplifier, which generates 120 fs, 800 nm laser pulses at a 1 kHz repetition rate. As depicted schematically in Figure 1a, the experimental configuration uses red lines for optical beams and blue regions for THz fields. The laser beam was split into three branches: (1) THz generation, (2) optical pumping, and (3) THz detection. A ZnTe crystal emitted a THz wave, which were focused onto the sample to characterize the transmission changes as a function of the optical pump delay. Laser excitation of NiFe films enabled the study of their THz emission spectroscopy. The optical pump was normally incident on the sample, and pump‐induced THz emission was detected after propagating through the Al_2_O_3_ substrate. The 800 nm optical pump beam was used without focusing, producing a 4 mm diameter spot on the sample. The THz probe beam was focused by a 15.2 cm‐focal‐length off‐axis parabolic mirror, forming a 2 mm diameter spot. The larger pump spot ensured uniform excitation across the entire THz probing region, eliminating spatial inhomogeneity effects in OPTP measurements. The parabolic mirrors with small apertures were used to transmit the optical beam while minimizing THz propagation losses. A chopper was placed in the optical pump path. The sample was placed in a static in‐pane magnetic field ± H  =  140 mT. The THz electric field was detected using an electro‐optical ZnTe detector. A THz‐wave‐transparent optical filter was employed to eliminate residual optical pulses. To prevent THz absorption by water molecules, the entire setup was enclosed in a dry‐air purge box. All measurements were performed at ambient temperature (see Section S8 for details).

### 4.3 Equations of Electron–Phonon Coupling

The energy exchange rate between electrons and phonons is characterized by the coupling constant *G*_ep_ calculated as [59]

(9) $$G_{\mathrm{ep}}=0.562n_{e}\frac{k_{B}^{2}\Theta_{D}^{2}v_{F}}{L_{F}T_{l}E_{F}}$$

where, *n*_e_, *E*_F_, Θ_D_, *v*_F_ and *L*_F_ represent the free electron density, Fermi energy, Debye temperature, Fermi velocity, and electron mean free path, respectively. Herein, the Fermi velocity can be expressed as:

(10) $$v_{F}=\frac{\hbar k_{F}}{m^{*}}$$

Fermi energy is expressed as:

(11) $$E_{F}=\frac{\hbar^{2}k_{F}^{2}}{2m^{*}}\;$$

Electron mean free path is expressed as:

(12) $$L_{F}=v_{F}\;\tau$$

Then, substituting Equations (10)–(12) into Equation (9) gives:

(13) $$G_{\mathrm{ep}}=2\times 0.562n_{e}\frac{k_{B}^{2}\Theta_{D}^{2}}{T_{l}}\frac{m^{*}}{\hbar^{2}k_{F}^{2}\tau }$$

Additionally, the Fermi wavenumber *k*_F_ can be expressed as:

(14) $$k_{F}={\left(3\pi^{2}n_{e}\right)}^{\frac{1}{3}}$$

and electron mean free time τ is expressed as

(15) $$\tau =\frac{m^{*}}{n_{e}e^{2}\rho }$$

Finally, substituting Equations (14) and (15) into Equation (13) yields:

(16) $$G_{\mathrm{ep}}\approx 0.1175{\left(\frac{k_{B}\Theta_{D}e}{\hbar }\right)}^{2}\frac{\rho }{T_{l}}n_{e}^{\frac{4}{3}}$$

## Funding

This work was supported by the National Key Research and Development Program of China (Grant No. 2023YFF0719200); the National Natural Science Foundation of China (Grant Nos. 62322115, U24A20226, 62588201, 62435010, 62335012, 62027807 and 12574133); Shanghai Educational Development Foundation (Grant Number. 24SG46); the 111 Project (Grant No. D18014); the Key project supported by Science and Technology Commission of Shanghai Municipality (Grant No. YDZX20193100004960); the Science and Technology Commission of Shanghai Municipality (Grant No. 22JC1400200); and the General Administration of Customs People's Republic of China (Grant No. 2019HK006) and the State Assignment of Lomonosov Moscow State University.

## Conflicts of Interest

The authors declare no conflicts of interest.

## Supporting information




**Supporting File**: advs75727‐sup‐0001‐SuppMat.docx.

## Data Availability

The data that support the findings of this study are available from the corresponding author upon reasonable request.
