# Supplementary material for: Ultrafast Dynamics of Spin Current and Electron Temperature in Spintronic Terahertz Emitters
Source: Adv Sci (Weinh). 2026 May 15:e75727. Online ahead of print. doi: 10.1002/advs.75727 (PMC13335852; doi:10.1002/advs.75727)
Supplement: Supplementary file 1 — Supporting File: advs75727‐sup‐0001‐SuppMat.docx. [file ADVS-9999-e75727-s001.docx]

**Support Information**

**Ultrafast dynamics of spin current and electron temperature in spintronic terahertz emitters**

*Yifan Wang*^†^*, Zuanming Jin^*，^*^†^*, Miao Cai, Zhiqiang Lan, Zhenjie Ge, Zheng Feng, Wei Tan^*^, Alexei V. Balakin, Alexander P. Shkurinov, Yan Peng^*^, and Yiming Zhu^*^*

Yifan Wang, Zuanming Jin, Miao Cai, Zhiqiang Lan, Zhenjie Ge, Yan Peng, Yiming Zhu

THz Technology Innovation Research Institute, THz Spectrum and Imaging Technology Cooperative Innovation Center, Shanghai Key Lab of Modern Optical System, University of Shanghai for Science and Technology, Shanghai 200093, China

E-mail: (physics_jzm@usst.edu.cn, py@usst.edu.cn, ymzhu@usst.edu.cn)

Zheng Feng, Wei Tan

Microsystem & Terahertz Research Center, CAEP, Chengdu 610200, People’s Republic of China

E-mail: (tanwei@mtrc.ac.cn)

Alexei V. Balakin, Alexander P. Shkurinov

Faculty of Physics, Lomonosov Moscow State University, Leninskie Gory 1, Moscow 19991, Russia

†These authors contributed equally.

**Supplementary Section 1**

**Figure S1**. THz-TDS of NiFe under $\pm$H, where the red solid line represents the spectrum under +H and the blue dashed line represent the spectrum under -H.

We measured the terahertz transmission spectra, namely terahertz time-domain spectra, of NiFe under ±H without laser excitation, respectively, as shown in Figure S1. It can be observed that the terahertz transmission spectra exhibited almost no variation when the magnetic field direction was reversed.

**Supplementary Section 2**

To distinguish the contributions of ultrafast demagnetization (UDM) and the anomalous Hall effect (AHE) to the THz emission in single NiFe layer, we prepared a set of NiFe films with thicknesses ranging from 5 to 23 nm, and performed the thickness dependent measurements under two different configurations, as shown in Fig. S2. The pump fluence was fixed at 1.42 mJ/cm^2^. Figure S2 (a) and (b) show the THz waveforms from the NiFe films under +H, pumped from the ferromagnetic side (F) and the substrate side (S), respectively.


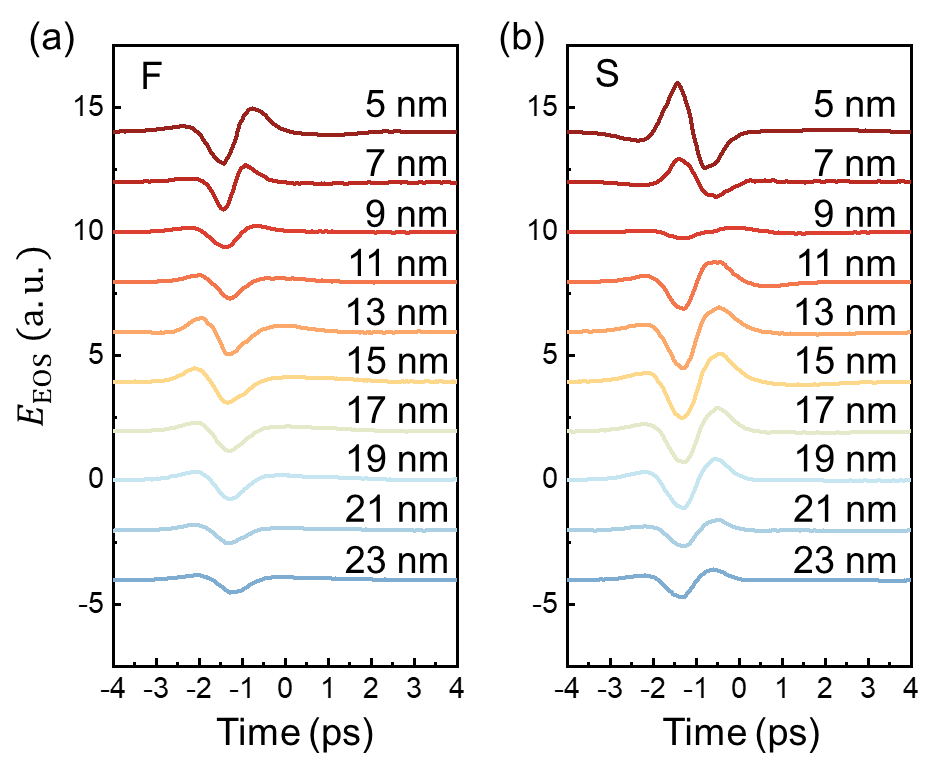


**Figure S2.** Thickness dependence of THz waveforms from NiFe single layers with +H at (a) F and (b) S. The THz waveforms are shifted in y axis for clarity.

The polarity of the THz emission by AHE is opposite for pumping from the substrate side (S) and from FM layer side (F). While, the UDM contribution does not change under the same magnetization. Thus, $\boldsymbol{E}_{\mathrm{EOS}}^{F}\left( t \right)=\boldsymbol{E}_{\mathrm{EOS}}^{\mathrm{UDM}}\left( t \right)+\boldsymbol{E}_{\mathrm{EOS}}^{\mathrm{AHE}}\left( t \right)$ and $\boldsymbol{E}_{\mathrm{EOS}}^{S}\left( t \right)=\boldsymbol{E}_{\mathrm{EOS}}^{\mathrm{UDM}}\left( t \right)-\boldsymbol{E}_{\mathrm{EOS}}^{\mathrm{AHE}}\left( t \right)$. Consequently, the distinct contributios of UDM- and AHE-based THz emission can be extracted by:

$$\boldsymbol{E}_{\mathrm{EOS}}^{\mathrm{UDM}}\left( t \right)=(\boldsymbol{E}_{\mathrm{EOS}}^{F}\left( t \right)+\boldsymbol{E}_{\mathrm{EOS}}^{S}\left( t \right))/2$$

$$\boldsymbol{E}_{\mathrm{EOS}}^{\mathrm{AHE}}\left( t \right)=(\boldsymbol{E}_{\mathrm{EOS}}^{F}\left( t \right)-\boldsymbol{E}_{\mathrm{EOS}}^{S}\left( t \right))/2$$

In Figure S3 (a) and (b), we decompose $\boldsymbol{E}_{\mathrm{EOS}}^{\mathrm{UDM}}\left( t \right)$ and $\boldsymbol{E}_{\mathrm{EOS}}^{\mathrm{AHE}}\left( t \right)$ waveforms for the NiFe films with different thickness. The peak amplitudes of $\boldsymbol{E}_{\mathrm{EOS}}^{\mathrm{UDM}}\left( t \right)$ and $\boldsymbol{E}_{\mathrm{EOS}}^{\mathrm{AHE}}\left( t \right)$ are plotted as functions of Fe thickness, as shown in Fig. S3 (c) and (d), respectively. For the 7 nm thickness NiFe, the contribution of AHE is 2.56 times that of UDM.

**Figure S3.** Thickness dependences of THz contributions from (a) UDM and (b) AHE mechanisms in NiFe single layers. The THz waveforms are shifted in y axis for clarity. The peak amplitudes of (c) $\boldsymbol{E}_{\mathrm{EOS}}^{\mathrm{UDM}}$ and (d) $\boldsymbol{E}_{\mathrm{EOS}}^{\mathrm{AHE}}$ are collected as functions of the NiFe thickness.

**Supplementary Section 3**

**Figure S4**. THz OPTP curves ${\Delta E}^{\pm H}(t)$ of NiFe film measured under $\pm$H with different pump fluences.

To further investigate both THz emission and carrier dynamics, we measured the THz OPTP curves under pump fluence ranging from 0.28 mJ/cm² to 1.42 mJ/cm², as shown in Figure S4. It can be observed that the THz OPTP curve increases with the rise of pump fluence.

**Supplementary Section 4**

**Figure S5**. **THz behaviors with respect to the fluences.** a) Measured THz waveforms

as a function of laser fluence from 0.28 to 1.42mJ/cm^2^. b) THz spectra corresponding to (a). c) Normalized THz spectra corresponding to (a). d) Peak frequency of THz emission as a function of pump fluence.

Figure S5a and S5b show the measured THz waveforms and the corresponding THz spectra of the THz emission with laser excitation varied from 0.28 to 1.42 mJ/cm^2^, respectively. It can be observed that the amplitude of terahertz waves increases with the rise of pump fluence. We normalized the THz spectra presented in Figure S5b, and the corresponding results are shown in Figure S5c. Meanwhile, the laser fluence dependence of the THz peak frequency is shown in Figure S5d. It is obvious that the peak frequency decreases from 0.6104 to 0.5082 THz when the laser fluence changes from 0.28 to1.42 mJ/cm^2^.

**Supplementary Section 5**

**Figure S6**. The experimental values of the transmittance, reflectance, and absorptance ratios at different laser fluences.

**Table S1** The experimental values of the transmittance, reflectance, and absorptance ratios at different laser fluences.

| Pump fluence (mJ/cm^2^) | Transmitted ratio (%) | Reflected ratio  (%) | Absorption ratio (%) |
| --- | --- | --- | --- |
| 0.28 | 28 | 27.67 | 44.43 |
| 0.57 | 27.17 | 33.33 | 39.5 |
| 0.85 | 25 | 29.44 | 45.56 |
| 1.13 | 24 | 28.67 | 47.33 |
| 1.42 | 22.93 | 28.47 | 48.6 |

We measured the transmitted, reflected, and absorbed power of the 7 nm NiFe thin film. Figure S6 presents the transmission coefficient, reflection coefficient, and absorption ratio of the 7 nm NiFe thin film under irradiation with an 800 nm femtosecond laser. Specific data are listed in Table S1.

**Supplementary Section 6**


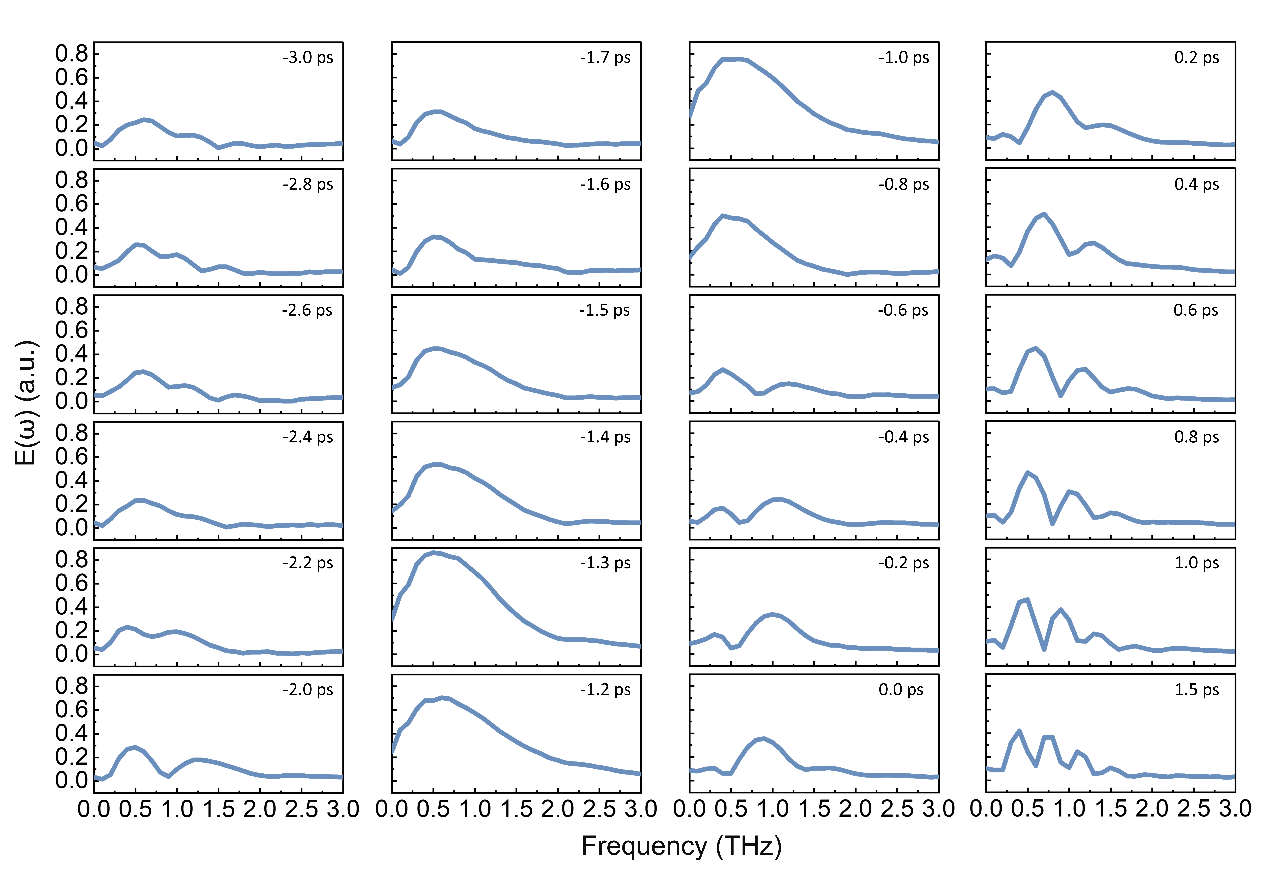


**Figure S7.** Terahertz spectra at different pump delays.

Figure S7 displays the THz spectra under different pump delay conditions ranging from a relative time of -3.0 ps to 1.5 ps. Two specific cases are highlighted in the spectral plots: first, when the probe pulse impinges before the system is excited, the overall spectrum is completely consistent with the terahertz radiation spectrum; second, when the probe pulse arrives after the system excitation, distinct interference peaks emerge in the spectrum.


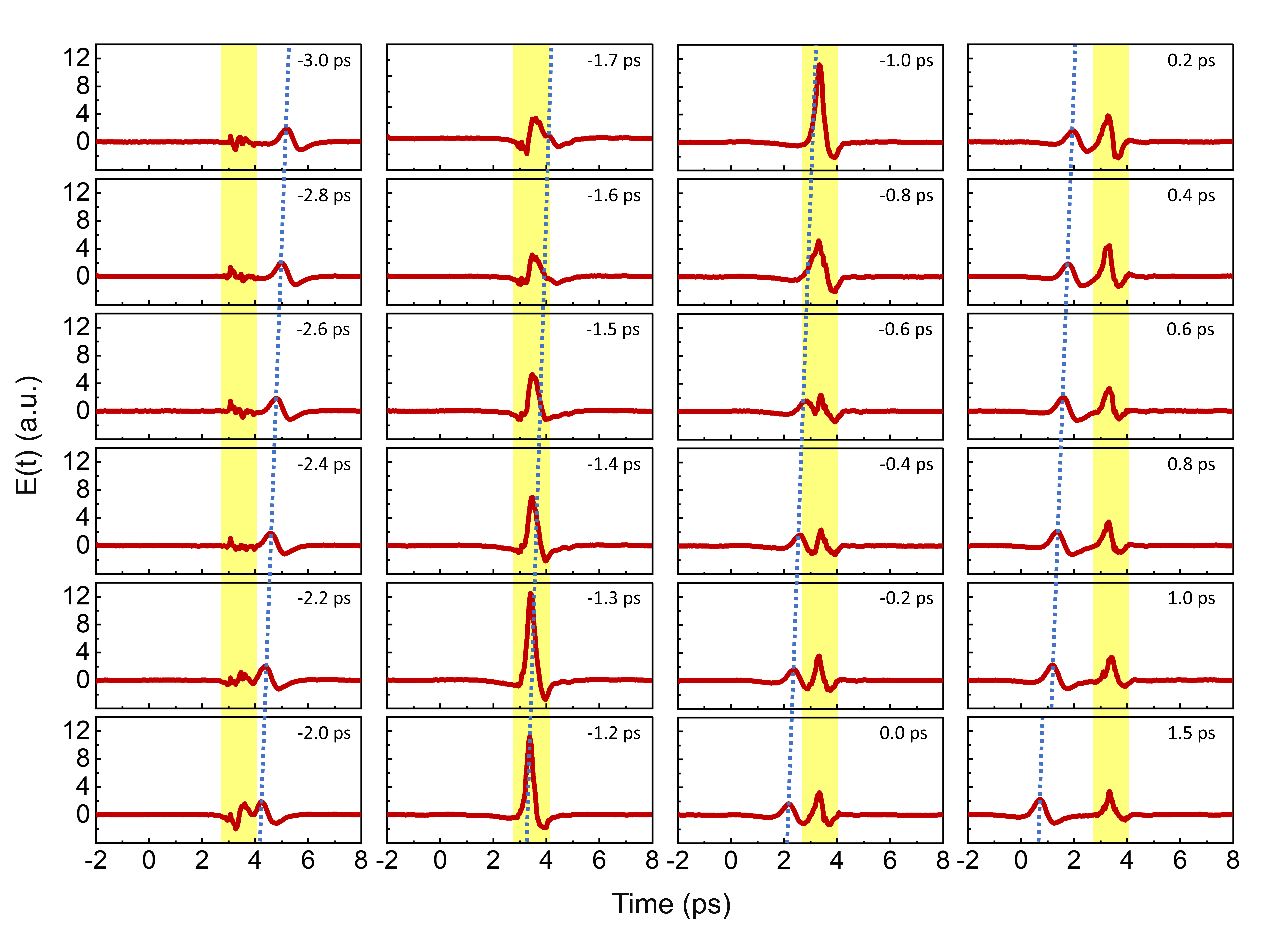


**Figure S8.** Terahertz time-domain spectra at different pump delays.

Figure S8 shows a view of the additional peak (highlighted in yellow) in the measured signal, along with a schematic representation of the different relative timing configurations of the optical pump pulses. When the optical pump pulse arrives after the terahertz probe pulse, shown in the left panel, no peak is visible. However, when the pump pulse excites the wafer before the terahertz probe pulse arrives, the additional peak appears in the time trace of the terahertz waveform, always at the same time delay as the terahertz probe pulse.

**Supplementary Section 7**

**Figure S9.** (a)-(f) THz spectra data at +0.2 ps, +0.4 ps, +0.6 ps, +0.8 ps +1.0 ps and +1.5 ps. (g)-(l) Associated THz-TDS data at +0.2 ps, +0.4 ps, +0.6 ps, +0.8 ps +1.0 ps and +1.5 ps.

First, in the experiment, a relative time position of $-$1.19 ps is recorded as the position of the THz detection pulse. This relative time position of the THz probe pulse is fixed. Second, the pump beam stepper motor is moved to the relative time positions of +0.2 ps, +0.4 ps, +0.6 ps, +0.8 ps +1.0 ps and +1.5 ps, giving the temporal delays of the THz probe relative to the optical pump pulse of Δt_1_=1.39 ps, Δt_1_=1.59 ps, Δt_1_=1.79 ps, Δt_1_=1.99 ps, Δt_1_=2.19 ps and Δt_1_=2.69 ps, respectively. The interference patterns of the THz generation and the THz probe pulses at six moments are recorded in Figure S9 (a)-(f), respectively.

A notable observation is that distinct interference peaks appear across the spectrum when the probe pulse is introduced after the pump pulse. The temporal gap Δt_2_ of THz radiation and the probe pulse can be derived from the inter-peak frequency difference Δf of the overall spectrum with the relationship approximated by Δt_2_$\approx\frac{1}{\Delta f}$. As shown in Figure S9 (g)-(l), the Δt_2_ are 1.32 ps, 1.53 ps, 1.73 ps, 1.92 ps, 2.12 ps, and 2.62 ps, respectively. Finally, we obtain the time lag between THz emission and laser excitation Δt at different relative delay times: 70 fs, 60 fs, 60 fs, 70 fs, 50 fs, and 70 fs, with an average value of approximately 63±8 fs.

To further verify the obtained time lag, we extracted the time lag of 7 nm NiFe at higher pump fluence of 1.98 mJ/cm² using the same method and procedures, as shown in Figure S10. We also observed a time lag of 63±5 fs, which is consistent with the result obtained at the pump fluence of 1.42 mJ/cm².

**Figure S10.** a) 2D spectral map of $E_{emit}+\Delta E$ vs. THz frequency and time delay, when the THz probe sent in the Al_2_O_3_/NiFe (7 nm)/SiO_2_ after the excitation of 1.98 mJ/cm². b) THz spectra and c) THz-TDS data at +0.6 ps relative time. d) The time lags between THz emission and laser excitation Δt measured at different relative delay times.

**Figure S11.** a) 2D spectral map of $E_{emit}+\Delta E$ vs. THz frequency and time delay, when the THz probe sent in the Al_2_O_3_/NiFe (3 nm)/SiO_2_ after the excitation of 1.42 mJ/cm². b) THz spectra and c) THz-TDS data at +0.6 ps relative time. d) The time lag between THz emission and laser excitation Δt at different relative delay times.

Furthermore, we performed supplementary experiments on 3 nm-thick NiFe alloy at an excitation fluence of 1.42 mJ/cm², and measured a time lag of approximately 61.7±7.5 fs, as shown in Figure S11.

**Supplementary Section 8**

**Optical pump THz probe spectroscopy.** The OPTP spectroscopy was performed using a Ti:sapphire regenerative amplifier, which generates 120 fs, 800 nm laser pulses at a 1 kHz repetition rate. As depicted schematically in Figure 1a, the experimental configuration uses red lines for optical beams and blue regions for THz fields. The laser beam is split into three branches: (1) THz generation, (2) optical pumping, and (3) THz detection. A ZnTe crystal emits THz wave, that are focused onto the sample to characterize the transmission changes as a function of the optical pump delay. Laser excitation of NiFe films enables the study of their THz emission spectroscopy.

The optical pump beam (800 nm) was used without focusing, yielding a spot diameter of 4 mm on the sample surface. The THz probe beam was focused by an off-axis parabolic mirror with a focal length of 15.2 cm, resulting in a spot diameter of 2 mm on the sample. The pump beam spot (4 mm) is significantly larger than the THz probe spot (2 mm), ensuring that the entire THz probing area is uniformly excited by the optical pump, thus eliminating any spatial inhomogeneity effects in the OPTP measurements.

The absolute values of the THz electric field can be calculated according to the current measurement from Lock-in amplifier as following:

$$\frac{\Delta V}{V_{0}}=\frac{\Delta I}{I_{probe}}=\frac{{\omega n}^{3}E_{THz}r_{41}L}{c}$$

Here, the nonzero electro-optic coefficient (ZnTe) $r_{41}$ = 3.9 pm/V, the refractive index of ZnTe in the infrared region $n$ =3. $c$ and $\omega$ are the speed of light in vacuum and the circularfrequency, and the length of the crystal $L$ = 1 mm. In the experiment, $V_{0}$ is 13.2 mV, and the peak amplitude of measured $\frac{\Delta V}{V_{0}}$ = 6.4×10^-2^; therefore, we can calculate the peak value of THz electric field $E_{THz}$ = 803 V/cm.

The peak amplitude of the intrinsically emitted THz field from the sample is much weaker than the incident THz probe field, with an amplitude ratio of approximately 1:63.8 under our experimental conditions, as shown in Figure S12.

**Figure S12.** Comparison between the emitted THz field (red) and the incident probe THz field (blue).
